# Gas permeability characteristics of the hanging wall and footwall in normal faults based on true triaxial experimental system

**DOI:** 10.1038/s41598-025-09179-5

**Published:** 2025-07-01

**Authors:** Weihua Song, Luo Yan, Huice Jiao

**Affiliations:** College of Mining, Liaoning Technical University, Fuxin, Liaoning China

**Keywords:** Coal and gas outburst, True triaxial experimental system, Normal fault, Permeability, Enhanced gas control, Energy science and technology, Solid Earth sciences, Fluid dynamics

## Abstract

In deep mining environments, normal fault structures significantly influence coal seam permeability, which plays a crucial role in coal and gas outbursts. Utilizing a true triaxial multifunctional physical simulation system, this study conducted gas seepage and hydraulic fracturing experiments on normal fault-associated coal seams at depths of − 400 m, − 600 m, and − 800 m. The research systematically investigates the gas permeability evolution in hanging wall and footwall coal seams of normal faults and proposes enhanced control measures tailored to the Guodishan normal fault. The results showed: Permeability exhibited a negative exponential relationship with confining pressure. As confining pressure increases, the permeability disparity between the hanging wall and footwall diminishes. Under true triaxial conditions, the permeability of the coal in the hanging wall of the normal fault is generally lower than that in the footwall. This difference gradually increases with burial depth, with the permeability ratio between the two sides increasing from 2.96 to 10.2 times. Moreover, the permeability reduction in the hanging wall coal is greater than that in the footwall coal. The crack initiation pressure for fracturing is higher in the hanging wall. Post-fracturing, permeability increases significantly in both walls, with the footwall demonstrating superior enhancement. However, the permeability amplification factor decreases progressively with depth. Building on the evolutionary characteristics of the Guodishan normal fault and its permeability patterns, targeted gas control strategies were developed in the Pingdingshan mining area. This work provides theoretical and practical guidance for gas extraction and outburst prevention in fault-affected coal seams.

## Introduction

Gas accidents, particularly coal and gas outbursts, represent one of the most severe safety hazards in coal mining operations. Research indicates that approximately 80% of outburst accidents occur in areas with active geological structures, and that the distribution and hazard of outbursts exhibit a highly regular association with these geological structures^1,2^. Faults, as common geological features, disrupt coal seam continuity and integrity through tectonic movements, leading to reduced mechanical strength, the formation of low-permeability tectonic coal, and impeded gas migration and dissipation. These processes result in localized accumulation of high gas content and pressure. Mining activities near fault zones significantly increase the risk of coal and gas outbursts^3^. Coal permeability, as one of the key factors in studying the mechanisms of coal and gas outbursts in fault-structure regions, holds significant guiding value for the identification, prediction, and prevention/control of such outbursts^4–6^.

Extensive research has been conducted by domestic and international scholars on the influence of fault structures on coal and gas outburst mechanisms, achieving notable results. Song et al.^7^ employed geo-dynamic division to analyze stress field distribution and outburst risks, revealing that superimposed fault structures govern the distribution of in-situ stress while spatially and temporally constraining outburst occurrences in fault-controlled zones. Regarding permeability and gas enrichment patterns, Gao et al.^8^ investigated the permeability characteristics of compression-torsion minor fault clusters using in-situ cross-layer borehole comparative methods. Their work demonstrated that such fault clusters exhibit impermeability, leading to localized gas hyperaccumulation and heightened outburst risks. Guo et al.^9^ conducted a comprehensive theoretical study on fault structures and outburst mechanisms. Utilizing methods from gas geology, structural physics, and rock mechanics, they analyzed the characteristics of coal seams in regions influenced by strong fault-induced stress fields, noting their low permeability, high gas content, and high gas pressure, and discussed the implications of these features for coal and gas outbursts. Numerical modeling advances include Li et al.^10^, who used COMSOL numerical simulation software to study the distribution of in-situ stress, gas pressure, and gas permeability in the working face near reverse fault structures. The results indicate that the closer the mining working face is to the fault zone, the higher the risk of coal and gas outbursts. However, this study did not conduct a comparative analysis from the perspective of different burial depths in the hanging wall and footwall. Cao et al.^11^ established normal fault models in FLAC^D^ to investigate stress evolution at 660 m, 800 m, and 1000 m depths. They identified stress concentration in the hanging wall during mining as a potential determinant of heightened outburst risks, yet failed to incorporate gas dynamics in their analysis.

Although existing studies have provided theoretical foundations for understanding coal and gas outburst mechanisms in fault zones, current research predominantly focuses on stress field distribution, theoretical analysis, numerical simulations, or single-side fault characteristics (hanging wall or footwall). There is still a lack of studies on the differences in permeability between the hanging wall and footwall coal and the evolution of these differences under the combined influence of complex stress fields and fluid pressures. Moreover, systematic investigations into the permeability evolution of hanging wall and footwall coal under laboratory conditions, particularly under true triaxial stress environments, are scarce. Therefore, conducting laboratory permeability tests on coal from both the hanging wall and footwall under true triaxial conditions to analyze the evolution of fracture and permeability under complex stress and fluid flow environments is of significant theoretical and practical importance.

This study focuses on the Guodishan normal fault in the Pingdingshan mining area. Through true triaxial permeability experiments on coal, the variation patterns of coal permeability in the hanging wall and footwall of normal faults are systematically investigated. The goal is to explore the controlling mechanism of permeability differences between the two fault blocks on coal and gas outbursts. Furthermore, targeted enhanced gas control strategies are proposed for mining operations affected by this normal fault structure, providing actionable solutions for gas disaster mitigation.

## Engineering geological conditions

**Fig. 1:**
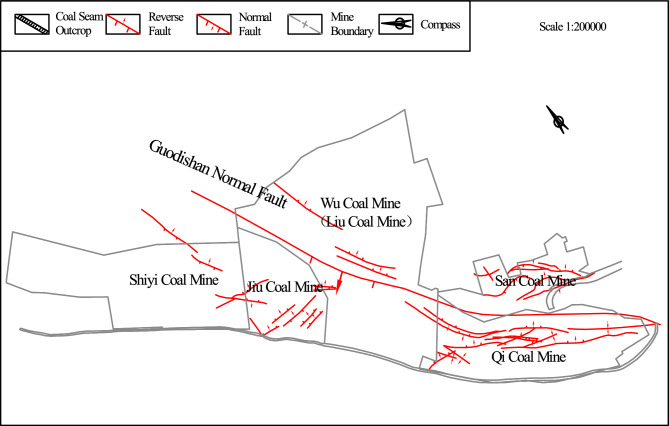
Position diagram of Guodishan fault.

The Guodishan normal fault is situated on the southwestern limb of the Likou Syncline, bounded by the Jiaxian Fault to the southeast and the Xiangjia Fault to the southwest (Fig. 1). This regional fault structure extends over 25 km from Shiyi Coal Mine through Jiu Coal Mine, Wu Coal Mine, Liu Coal Mine, San Coal Mine, and Qi Coal Mine to downtown Pingdingshan, critically influencing gas occurrence patterns in central and western mining districts. The fault exhibits a NW–SE trending orientation with a SW-dipping plane (dip angle: 45°-75°), displaying listric geometry characterized by steeper upper segments (65°) transitioning to gentler lower Sects. (30°). Stratigraphic analysis reveals vertical displacements of 100–220 m, where the NE block constitutes the footwall and the SW block forms the hanging wall. These structural attributes classify it as a high-angle normal fault system^12^.

### Tectonic evolution of the Guodishan normal fault

According to Anderson’s classical fault model^13^, fault formation results from the combined action of horizontal tectonic stress and vertical stress. Normal faults develop when the maximum principal stress (*σ₁*) acts in the vertical direction as a compressive stress, whereas reverse faults form when *σ₁* acts in the horizontal direction under horizontal compressive stress conditions.

**Fig. 2:**
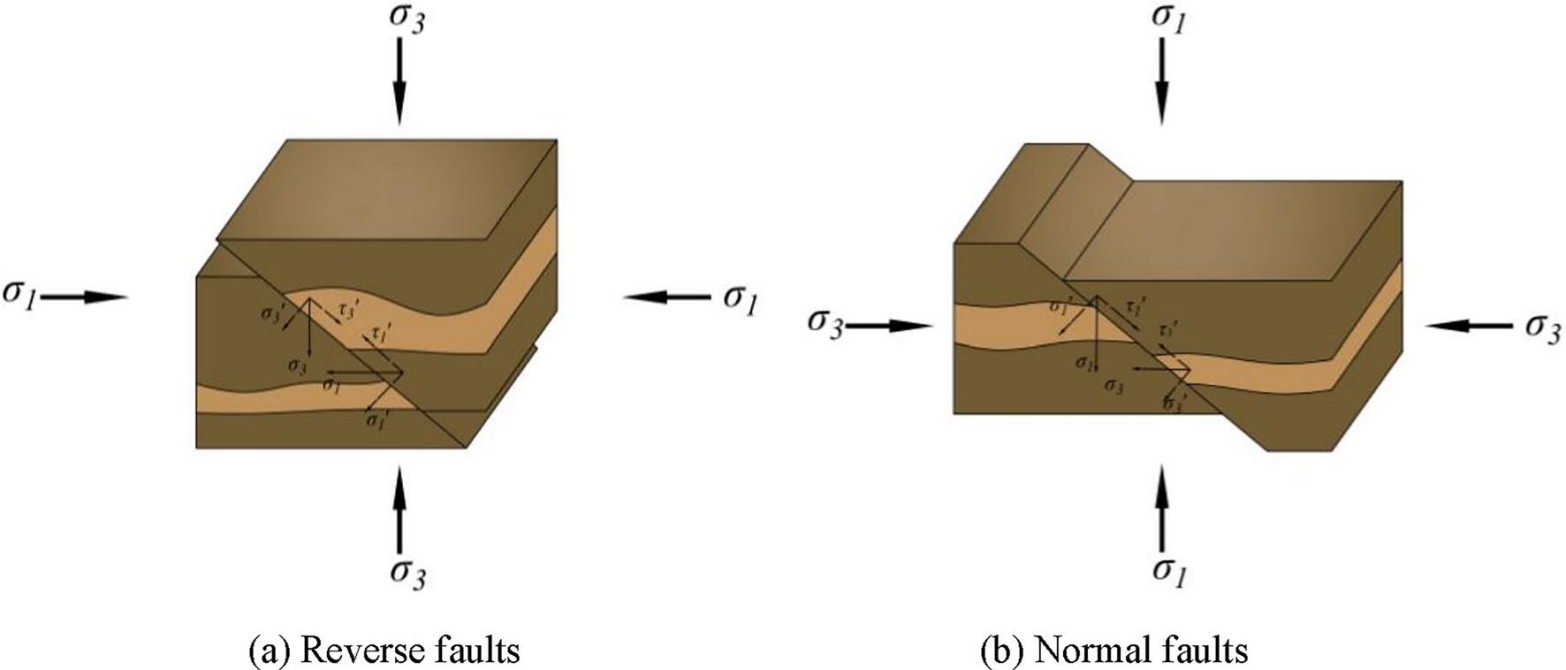
Mechanical mechanism of fault formation.

Reverse faults develop under a stress system dominated by horizontal tectonic stress, where the stratum undergoes conjugate shear fracturing. Under the continuous influence of tectonic stress, a fault plane develops, dividing the stratum into two parts: the upper part is the hanging wall, and the lower part is the footwall. The hanging wall acts as the active block during tectonic movement, where the horizontal shear stress component along the fault plane overcomes the downward shear stress component from vertical stress, causing the hanging wall to ascend along the fault surface. Conversely, the footwall remains relatively stable as the passive block, despite localized deformation near the fault zone, as illustrated in Fig. 2a. In contrast, normal faults form under vertical stress-dominated regimes. The vertical stress-induced fracture plane divides the stratum, with the hanging wall subsiding along the fault surface to create the characteristic normal fault structure, shown in Fig. 2b.

**Fig. 3:**
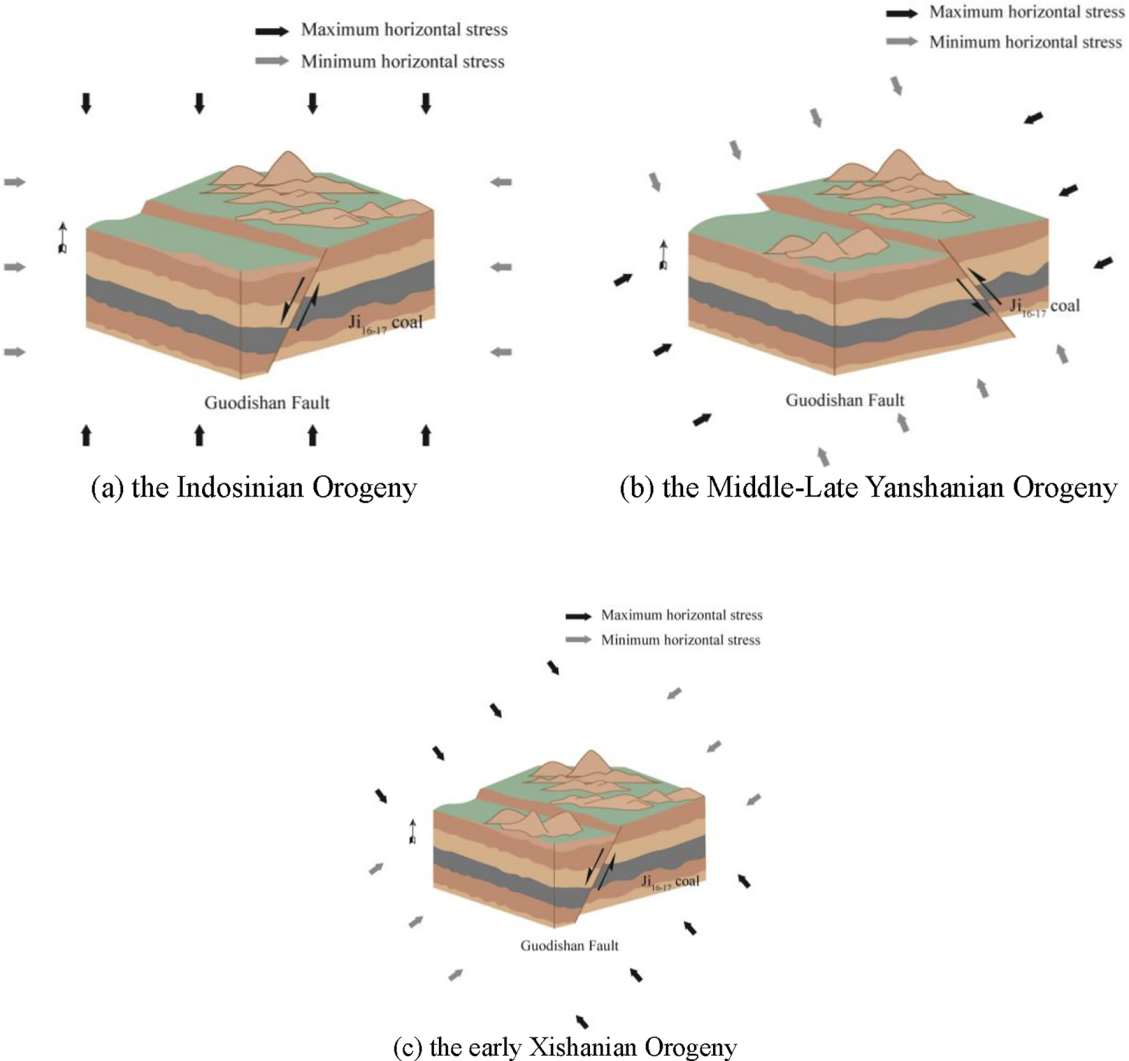
Tectonic evolution of the Guodishan fault.

The Guodishan fault initiated activity during the coal-accumulating period, controlling coal-bearing deposition and peat accumulation across its dual walls. Its most intense activity occurred in the Early Permian, exhibiting normal fault characteristics. During the Indosinian Orogeny, the North China stratum was significantly affected by tectonic activity, leading to intense folding, uplift, and faulting. The regional stress field was a nearly SN-oriented horizontal tectonic stress field, marking the first phase of the tectonic stress field since the Meso-Cenozoic era. However, the tectonic movement during this period did not cause deformation of the Guodishan fault structure, which remained a normal fault (Fig. 3a). In the Middle-Late Yanshanian Orogeny, the tectonic movement of the Qinling orogenic belt reached its peak, with the tectonic stress field primarily characterized by nearly NNE-SSW compressional forces. Under this compression, the Pingdingshan coalfield experienced near NNE-SSW-directed compressive stress, causing the southwestern (hanging wall) block of the Guodishan fault to thrust northeastward, forming a compressive reverse fault structure (Fig. 3b). During the early Xishanian Orogeny, the tectonic stress field was primarily characterized by nearly horizontal compression in the NWW-SEE direction and horizontal extension in the NNE-SSW direction. During this period, the Guodishan reverse fault, which had formed during the Yanshanian Orogeny, transformed into a normal fault. (Fig. 3c). From the Miocene to present, the tectonic stress manifested as an extrusive force on the Chinese mainland, and the Pingdingshan mining area experienced this extrusion. However, during this phase of tectonic movement, the deformation of the strata was relatively weak, and the Guodishan fault retained its normal fault configuration^14,15^.

The Guodishan normal fault originated as a syn-sedimentary normal fault during the coal-forming period. It transitioned into a reverse fault during the Middle-Late Yanshanian Orogeny and subsequently reverted to its current normal fault configuration in the early Xishanian Orogeny. The alternating compressional and extensional stresses within the fault zone caused varying degrees of damage to the coal seams. This normal-reverse-normal tectonic evolution significantly intensified coal fragmentation and enhanced the development of tectonic coal within the fault zone. Particularly in the hanging wall, which acted as the active block during tectonic movements, the coal experienced greater shear stresses, resulting in a higher degree of tectonic coal formation.

## Test device and scheme

### Test device

**Fig. 4:**
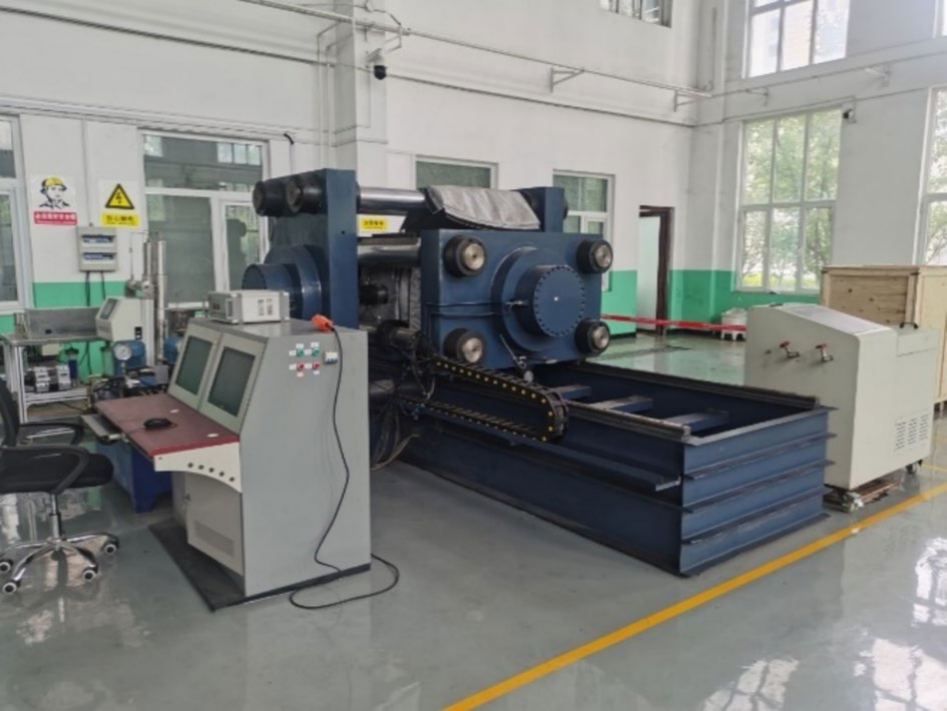
True triaxial multifunctional physical simulation system.

The seepage experiments were conducted using the true triaxial multifunctional physical simulation system (Fig. 4) at the State Key Laboratory of Coking Coal Resource Development and Comprehensive Utilization under Pingmei Shenma Group. This system enables gas adsorption/desorption, seepage, and hydraulic fracturing experiments under true triaxial stress conditions. The apparatus consists of a pressure vessel, a servo control system, a high-pressure seepage system, a fracturing system, and a data acquisition system, with a maximum confining stress capacity of 30 MPa and a gas injection pressure of 20 MPa.

**Fig. 5:**
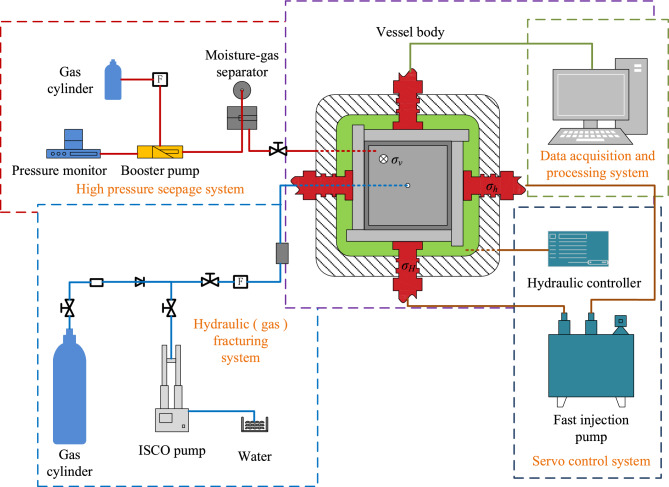
Schematic diagram of the experimental setup.

The pressure vessel serves as the container for specimen placement and hydraulic pressure bearing. The servo control system primarily utilizes a high-pressure plunger pump to inject and maintain hydraulic pressure within the vessel, applying axial stress (*σ₃*) to the specimen via pressurized water, with a maximum hydraulic pressure of 20 MPa. Horizontal stresses (*σ₁* and *σ₂*) are imposed on the specimen through hydraulic loading rods. The high-pressure seepage system conducts in-situ seepage experiments and permeability measurements under in-situ stress conditions. The fracturing system employs ISCO pumps to perform hydraulic fracturing operations. The data acquisition system integrates sensor data for real-time monitoring, storage, and export through dedicated software. A schematic diagram of the experimental setup is shown in Fig. 5.

Current laboratory investigations of gas permeability in coal predominantly utilize steady-state and transient methods to determine gas flow characteristics. It is widely accepted that gas migration through coal matrices adheres to Darcy’s law. Permeability variations during gas seepage directly reflect dynamic changes in the coal’s effective porosity, where increased permeability corresponds to enhanced pore connectivity^16^ This study employed the steady-state method to evaluate coal permeability under true triaxial stress conditions. Assuming Darcy-compliant gas flow through coal specimens, the permeability *k* (mD) of gas-saturated coal is calculated as^17^:

1 $$k = \frac{{2QP_{0} \mu L}}{{A(P_{1}^{2} - P_{2}^{2} )}}$$

where, *Q* is the volumetric flow rate (m^3^/s); *μ* is the gas dynamic viscosity (Pa·s); the seepage gas used in this experiment is high purity nitrogen, and the viscosity of nitrogen is 1.087 × 10^–6^ Pa·s at room temperature of 20 °C; *A* is the cross-sectional area (m^2^); *P*_*1*_ is coal the sample inlet gas pressure (Pa); *P*_*2*_ is the coal sample outlet gas pressure (Pa); *P*_*0*_ is the atmospheric pressure (0.1MPa); *L* is the specimen length (m).

### Specimen preparation

**Fig. 6:**
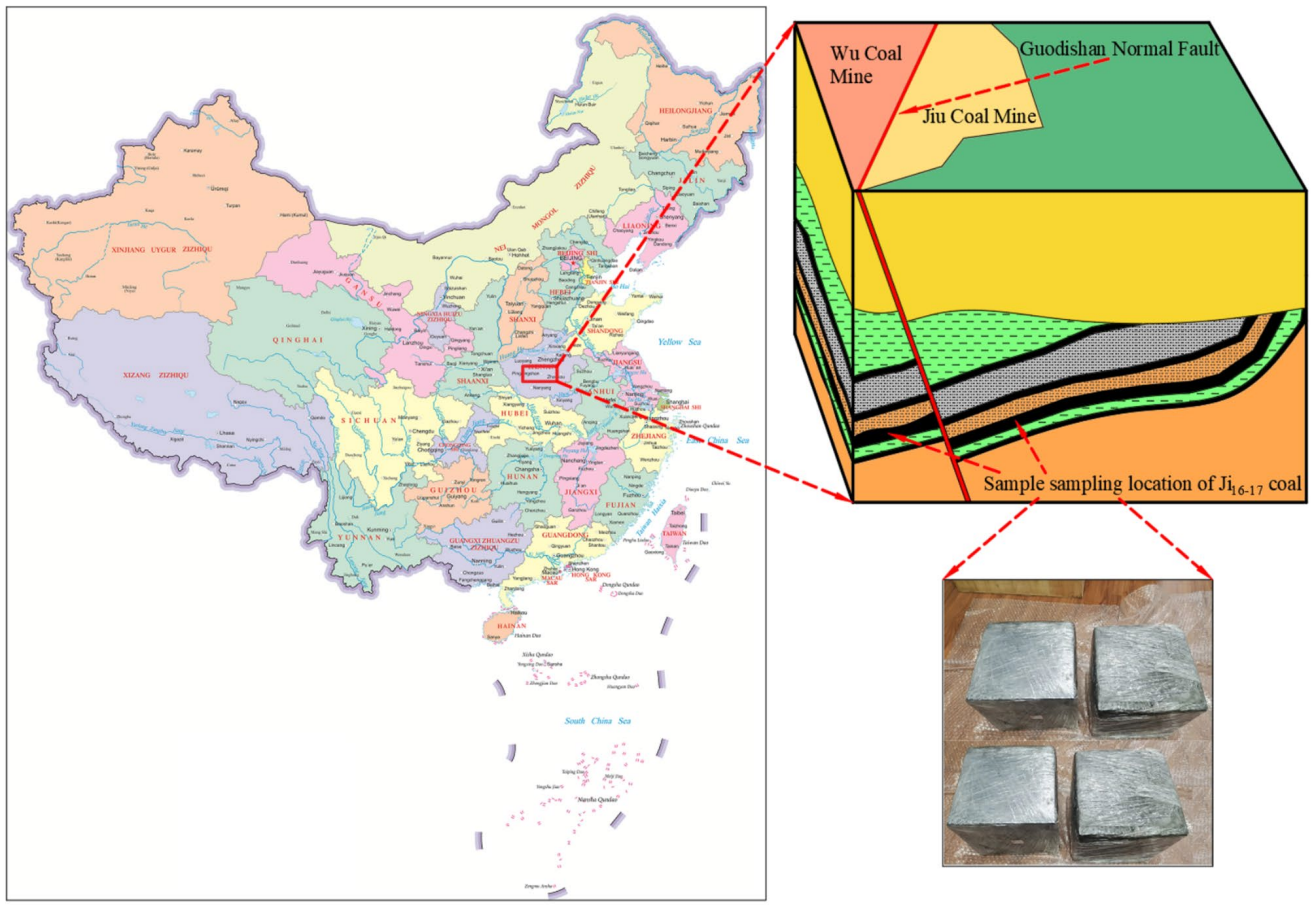
Geographical sampling locations of the coal seam and cubic specimens.

**Fig. 7:**
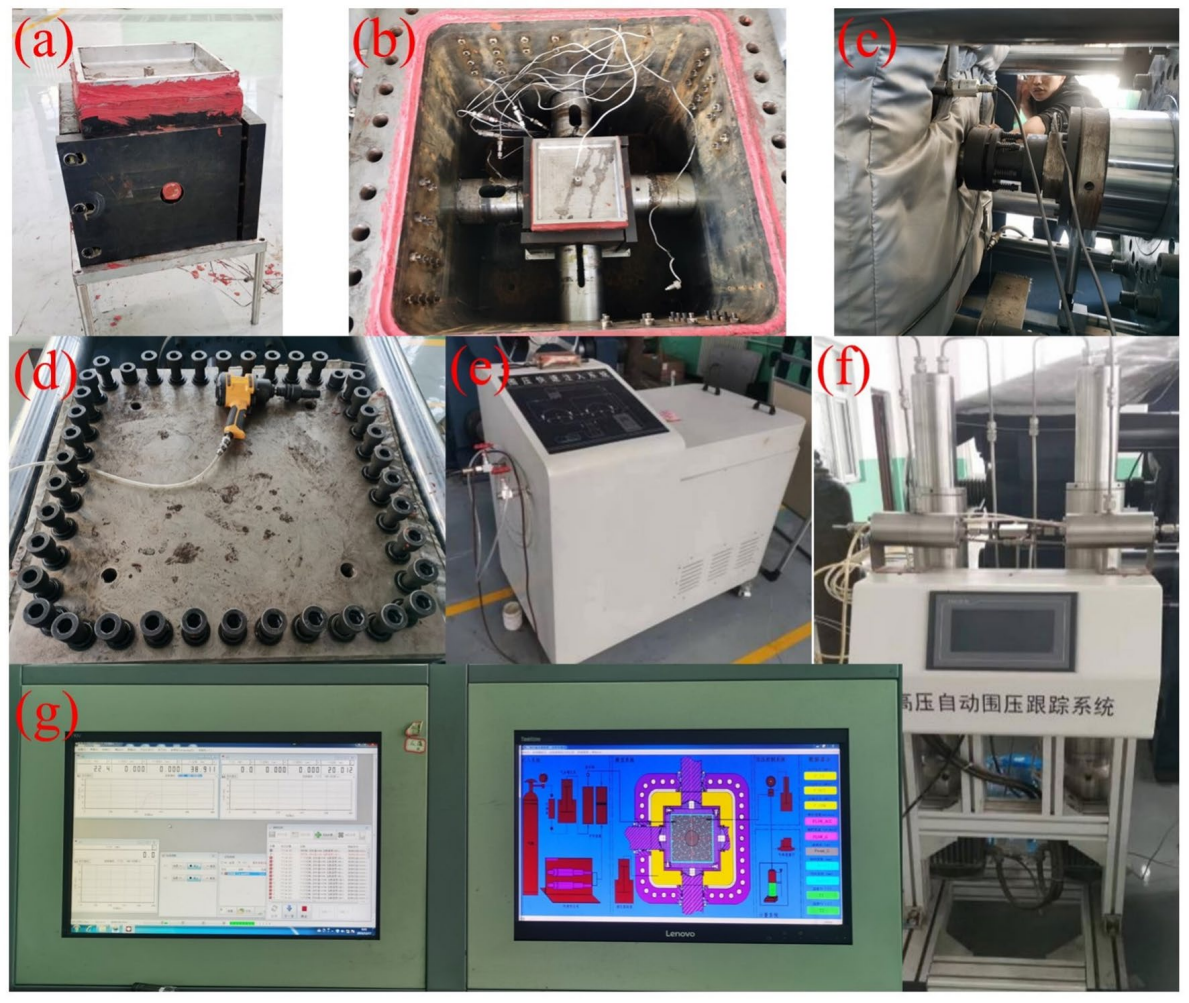
Experimental step diagram. (**a**) Sealing sample (**b**) Placing sample (**c**) Calibrate loading axis (**d**) Close the vessel (**e**) Pressure rapid injection system (**f**) Pressure tracking system (**g**) Permeability test.

The coal specimens were collected from the Ji_16-17_ coal seam in the hanging wall (Jiu Coal Mine) and footwall (Wu Coal Mine) of the Guodishan normal fault at this true triaxial permeability experiment (Fig. 6). The raw coal samples, with a density of 1.723 g/cm^3^, Young’s modulus *E* = 3.52 GPa, and Poisson’s ratio *ν* = 0.265, were sealed immediately after extraction from newly exposed coal faces and transported to the laboratory. The coal was crushed into particles, sieved to 0.18–0.38 mm, and mixed with purified water equivalent to 10% of the coal powder weight. The mixture was compacted in a briquette mold under an axial load of 800 kN (maintained for 30 min) using a rigid hydraulic press, producing 200 mm cubic samples. The average porosity of the coal briquette used in the experiment was 7.42%, while that of the raw coal from the sampling site was 5.71%. The porosity of the coal briquette was only 1.3 times that of the raw coal, indicating that the influence of the original coal structure on the experimental results is relatively limited^18^. After demolding, the coal specimens were dried in an oven for 24 h to remove inherent moisture and vacuum-sealed with preservative film (Fig. 7).

### Test procedure and scheme

Permeability testing procedure: the coal specimen was mounted in a rubber sleeve, followed by installation of the top cover, seepage pipes, and end plugs. Red sealant was applied to ensure airtightness, and loading plates were attached after the sealant cured. The assembly was hoisted into the pressure vessel, with precise alignment of loading axes and connection of seepage pipelines. The vessel was filled with water to the uppermost port level, after which the lid was secured and bolts tightened. The vessel was then positioned within the loading frame, engaging hydraulic rods with loading axes and connecting external gas lines. The confining pressure rapid injection system was activated to pressurize the vessel to 2 MPa. Subsequently, the confining pressure tracking system and lateral loading system were initiated, following predefined loading paths. Upon reaching target pressure levels, permeability measurements were conducted (Fig. 7). After each test cycle, the specimen was extracted, and the procedure repeated for subsequent samples.

**Fig. 8:**
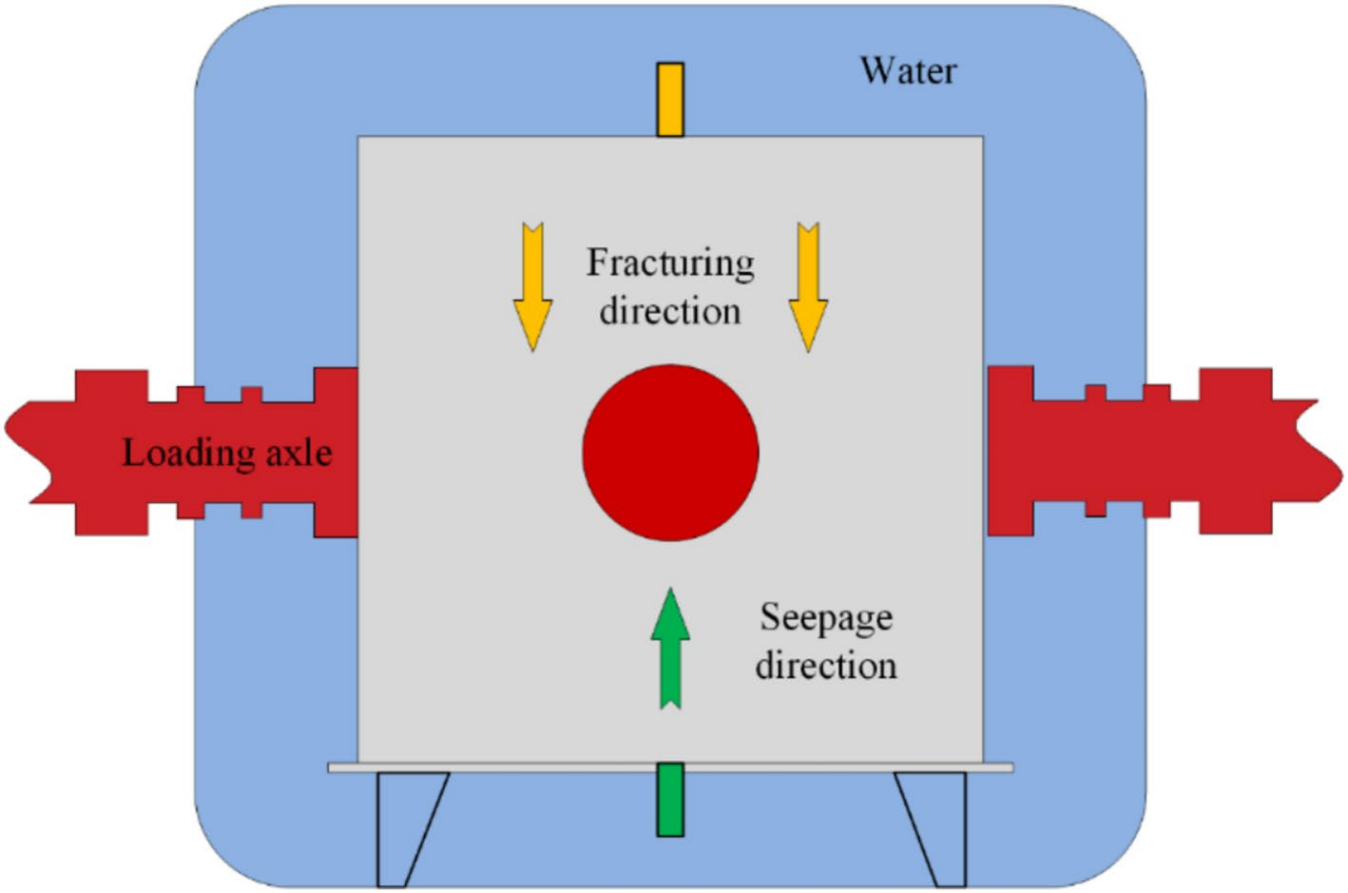
Schematic diagram of simultaneous seepage process of fracturing.

Hydraulic fracturing-integrated permeability testing procedure: a central fracturing borehole (*φ*10 mm × 90 mm) was drilled into the prepared coal specimen, followed by installation of a fracturing tube secured with sealant. After curing, the seepage pipeline configuration was modified to enable simultaneous fracturing and seepage (Fig. 8). Subsequent procedures mirrored standard permeability testing. Under true triaxial stresses replicating in situ conditions of the Guodishan fault walls, the fracturing system valve and seepage valve were opened. Hydraulic fracturing was conducted via an ISCO pump in constant-flow mode (60 mL/min). A pressure transducer at the fracturing tube outlet recorded real-time injection pressures; fracturing was deemed complete upon pressure stabilization. Permeability measurements were conducted concurrently with the fracturing process, following the identical testing protocol employed in pre-fracturing coal specimen permeability experiments.

### Test stress loading path

**Fig. 9:**
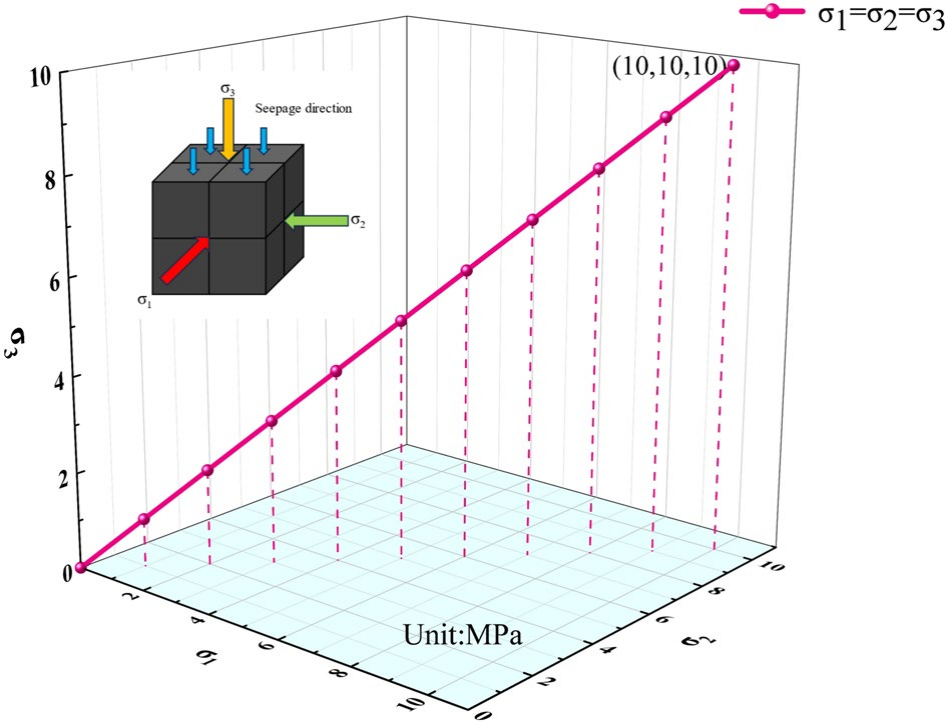
Stress loading path I.

**Fig. 10:**
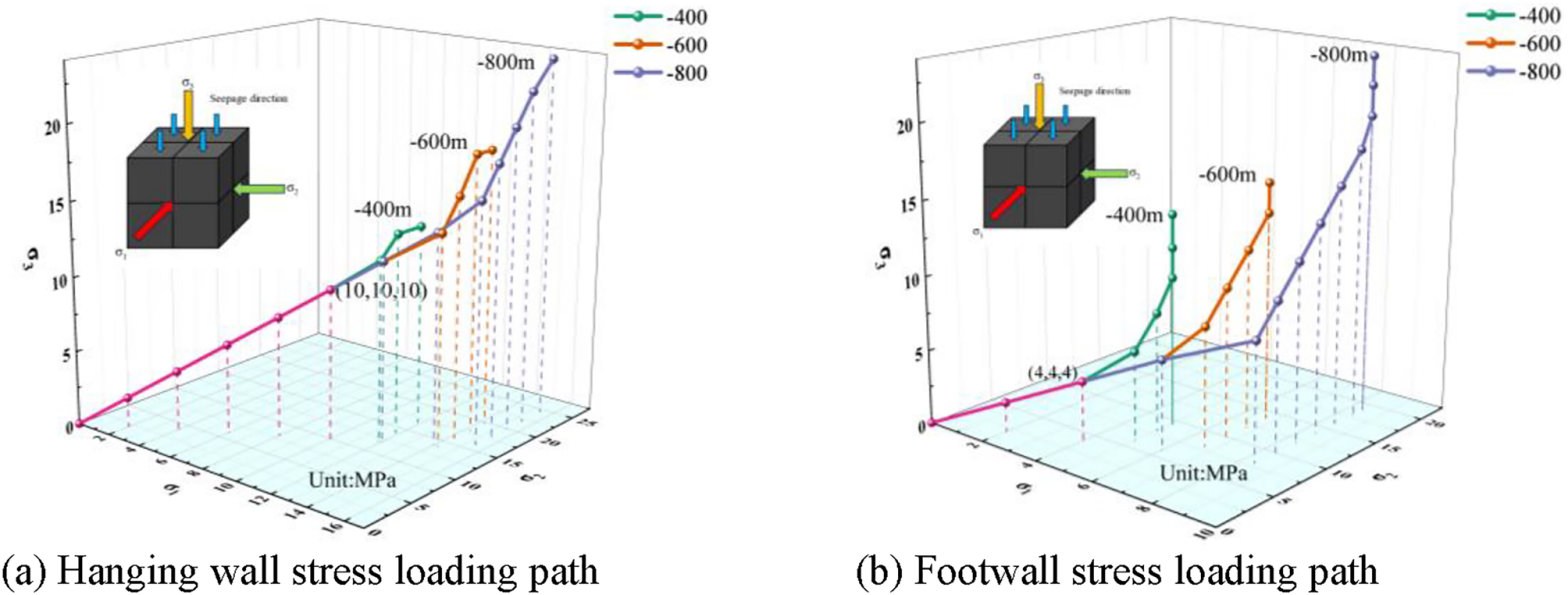
Stress loading path II.

The permeating gas used in the experiments was high-purity nitrogen (99.9%), applied at a gas pressure of 0.5 MPa. In this experiment, *σ*_*1*_, *σ*_*2*_, and *σ*_*3*_ were applied at a loading rate of 0.01 MPa/step. To investigate the intrinsic permeability characteristics of coal in the hanging wall and footwall of the Guodishan normal fault under equivalent stratigraphic elevation and in-situ stress conditions, two stress loading paths were designed based on the fault’s geostress distribution and geological context (Figs. 9 and 10).

To more accurately reflect the permeability variation of coal bodies at different elevations on the hanging wall and footwall of the Guodishan normal fault, we conducted in-situ stress measurements at sampling working faces located at -400 m, -600 m, and -800 m elevations on both sides of the fault. The original strain data obtained were processed and calculated, and the in-situ stress measurement results are shown in Table 1.

**Table 1:** In-situ stress loading table.

Elevation /(m)	State	Hanging wall			Footwall		
		*σ*_*H*_/(MPa)	*σ*_*v*_ /(MPa)	*σ*_*h*_ /(MPa)	*σ*_*H*_/(MPa)	*σ*_*v*_ /(MPa)	*σ*_*h*_ /(MPa)
− 400	In-situ stress	16.71	13.35	11.82	9.42	14.19	5.05
− 600		19.83	18.36	14.23	13.93	18.98	6.71
− 800		24.47	23.79	15.41	19.02	23.87	8.37

Stress Path I: Water was injected into the pressure vessel to establish an initial hydraulic pressure of 2 MPa (*σ₁* = *σ₂* = *σ₃* = 2 MPa); The confining pressure was then incrementally increased to 10 MPa following the stepwise loading trajectory illustrated in Fig. 9. Permeability tests were conducted at each integer stress level during the loading process.

Stress Path II: The pressure vessel was filled with water to establish an initial hydraulic pressure of 2 MPa. For coal specimens from the hanging wall of the Guodishan normal fault, in-situ stress corresponding to sampling elevations of − 400 m, − 600 m, and − 800 m (Table 1) were applied following the loading trajectory in Fig. 10a. Permeability tests were conducted upon reaching each target elevation. Similarly, for footwall coal specimens, in-situ stress at elevations of − 400 m, − 600 m, and − 800 m (Table 1) were imposed according to the stress path in Fig. 10b, with permeability measurements performed at each designated elevation.

## Results and analysis

**Fig. 11:**
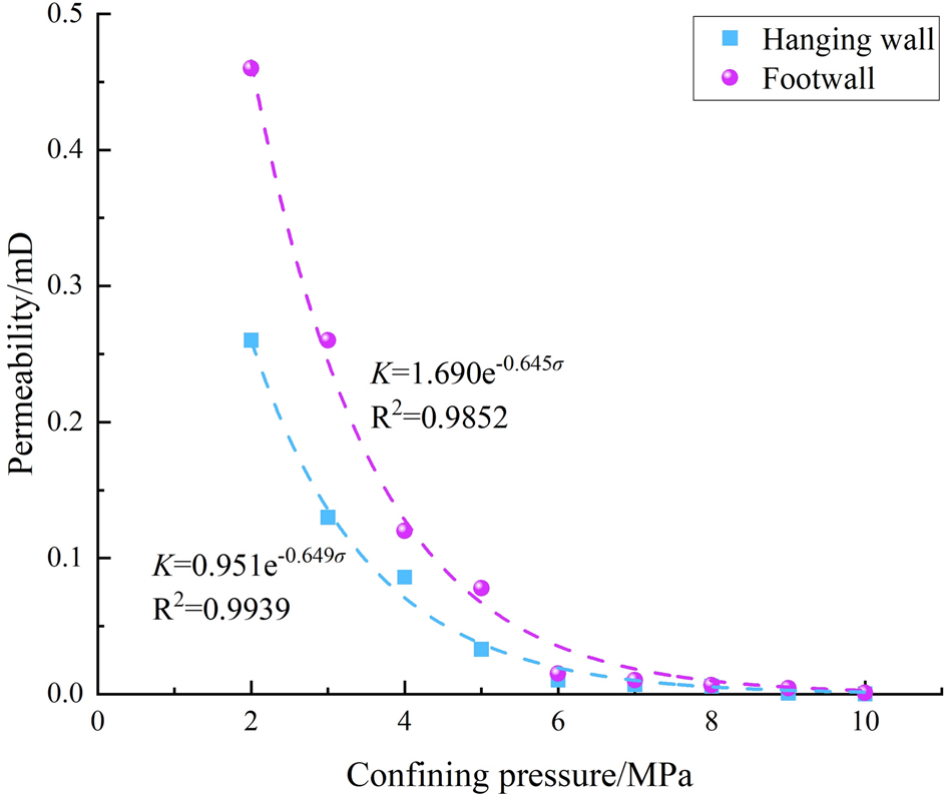
Evolution of coal permeability with confining pressure.

Figure 11 illustrates the permeability evolution and fitting curves of coal masses in the hanging wall and footwall of the normal fault under Stress Path I, demonstrating the relationship between permeability and confining pressure.

As shown in Fig. 11, the permeability of the coal in both the hanging wall and footwall decreases gradually with increasing confining pressure. Taking the hanging wall coal as an example, when the confining pressure increases from 2 to 10 MPa, the permeability decreases from 0.26 mD to 0.0002 mD, with a total reduction of 0.2598 mD. Specifically, when the confining pressure increases from 2 to 6 MPa, the permeability drops by 0.2497 mD, accounting for 96.11% of the total reduction. In contrast, as the confining pressure increases from 6 to 10 MPa, the permeability reduction accounts for only 3.89%. This indicates that the relationship between confining pressure and permeability is nonlinear. Through the fitting analysis of permeability and confining pressure, the two exhibit an approximate negative exponential relationship. The fitting equations for both sides of the normal fault are presented in Eqs. (2) and (3), with goodness-of-fit values of 0.9939 and 0.9852, respectively.

2 $${\text{Hanging wall:}}\;\;K_{U} = 0.951e^{ - 0.649\sigma }$$

3 $${\text{Footwall:}}\;\;K_{D} = 1.690e^{ - 0.645\sigma }$$

Under the same confining pressure conditions, the permeability of the coal in the hanging wall of the normal fault is lower than that in the footwall (Fig. 11). When the confining pressure reaches 2 MPa, the permeability of the hanging wall coal is 0.26 mD, while that of the footwall is 0.46 mD, with the hanging wall being 43.5% lower than the footwall. As the confining pressure increases to 6 MPa, the permeability of the hanging wall coal decreases to 0.0103 mD, while that of the footwall drops to 0.015 mD, making the hanging wall 31.3% lower. When the confining pressure reaches 10 MPa, the permeability of the hanging wall coal further declines to 0.0002 mD, while the footwall permeability is 0.00026 mD, with a difference of 23.1%. These results indicate that as the confining pressure increases, the permeability difference between the hanging wall and footwall gradually decreases.

**Fig. 12:**
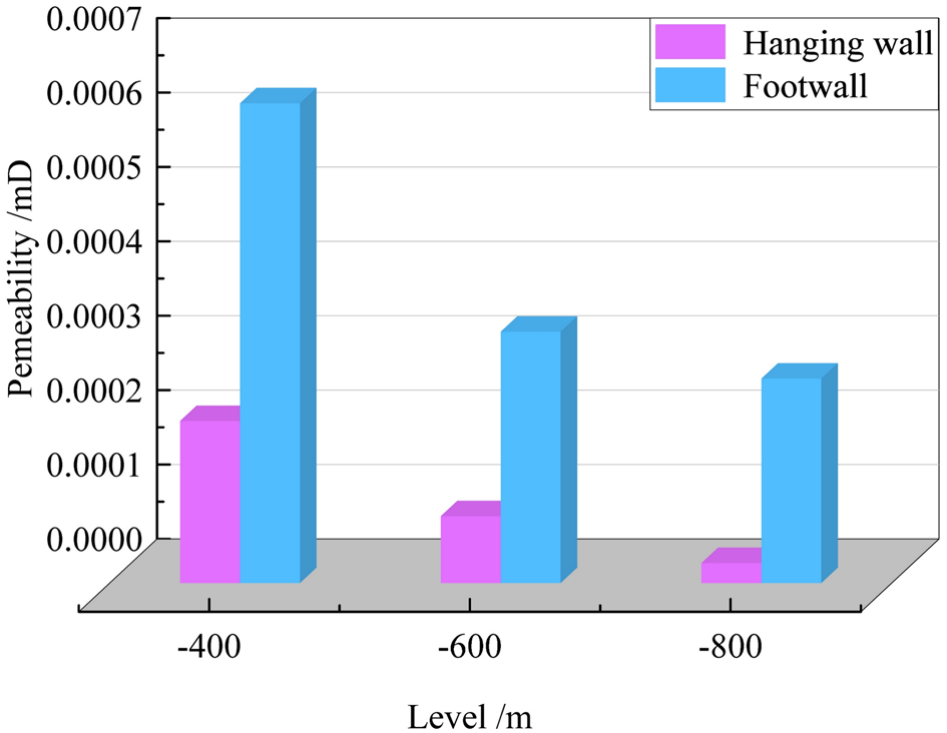
Permeability variation under in-situ stress condition.

Figure 12 presents the permeability test results of the hanging wall and footwall coal under true triaxial stress conditions along loading path II in the normal fault.

As shown in Fig. 12, under true triaxial stress conditions, the permeability of the hanging wall in the normal fault is generally lower than that of the footwall. At an elevation of -400 m under the initial in-situ stress conditions, the permeability of the hanging wall coal is 0.000218 mD, which is lower than that of the footwall at the same depth. As the burial depth increases, the permeability difference between the footwall and hanging wall coal increases significantly. At an elevation of -400 m, the permeability of the footwall is 2.96 times that of the hanging wall; at -600 m, this ratio increases to 3.75; and at -800 m, it reaches 10.2. This phenomenon indicates that the hanging wall coal of the normal fault provides better conditions for gas accumulation. However, with increasing burial depth, the permeability advantage of the footwall coal becomes more pronounced, and this difference is more significant in deeper coal seams.

Under true triaxial stress conditions, when the depth decreases from -400 m to -600 m, the permeability of the hanging wall coal decreases from 0.000218 mD to 0.00009 mD, a reduction of 58.7%. As the depth further decreases from -600 m to -800 m, the permeability drops to 0.000027 mD, representing a 70% reduction. In contrast, the permeability of the footwall coal decreases from 0.000645 mD at -400 m to 0.000338 mD at -600 m, a reduction of 47.6%, and further declines to 0.000275 mD at -800 m, a reduction of only 18.6%. This indicates that with increasing burial depth, the permeability of coal in both the hanging wall and footwall decreases, but the reduction in the hanging wall is more significant. Particularly from -600 m to -800 m, the permeability reduction reaches 70% in the hanging wall, whereas it is only 18.6% in the footwall. We think, this phenomenon can be attributed to the unique evolutionary history of the Guodishan Fault, which has undergone normal-reverse-normal transformations. This geological evolution has led to well-developed tectonic coal in the normal fault zone, with the hanging wall coal exhibiting lower permeability and better gas preservation conditions, resulting in higher gas pressure. Additionally, tectonic coal is characterized by low strength, easy fragmentation, and weak resistance, making it highly susceptible to failure and displacement, which can lead to coal and gas outbursts^19^. The presence of the Guodishan normal fault significantly affects the permeability of the hanging wall coal. While it may provide favorable conditions for gas accumulation, gas drainage is more challenging. In contrast, gas in the footwall coal flows more easily, resulting in lower gas content but making gas extraction and control more effective.

**Fig. 13:**
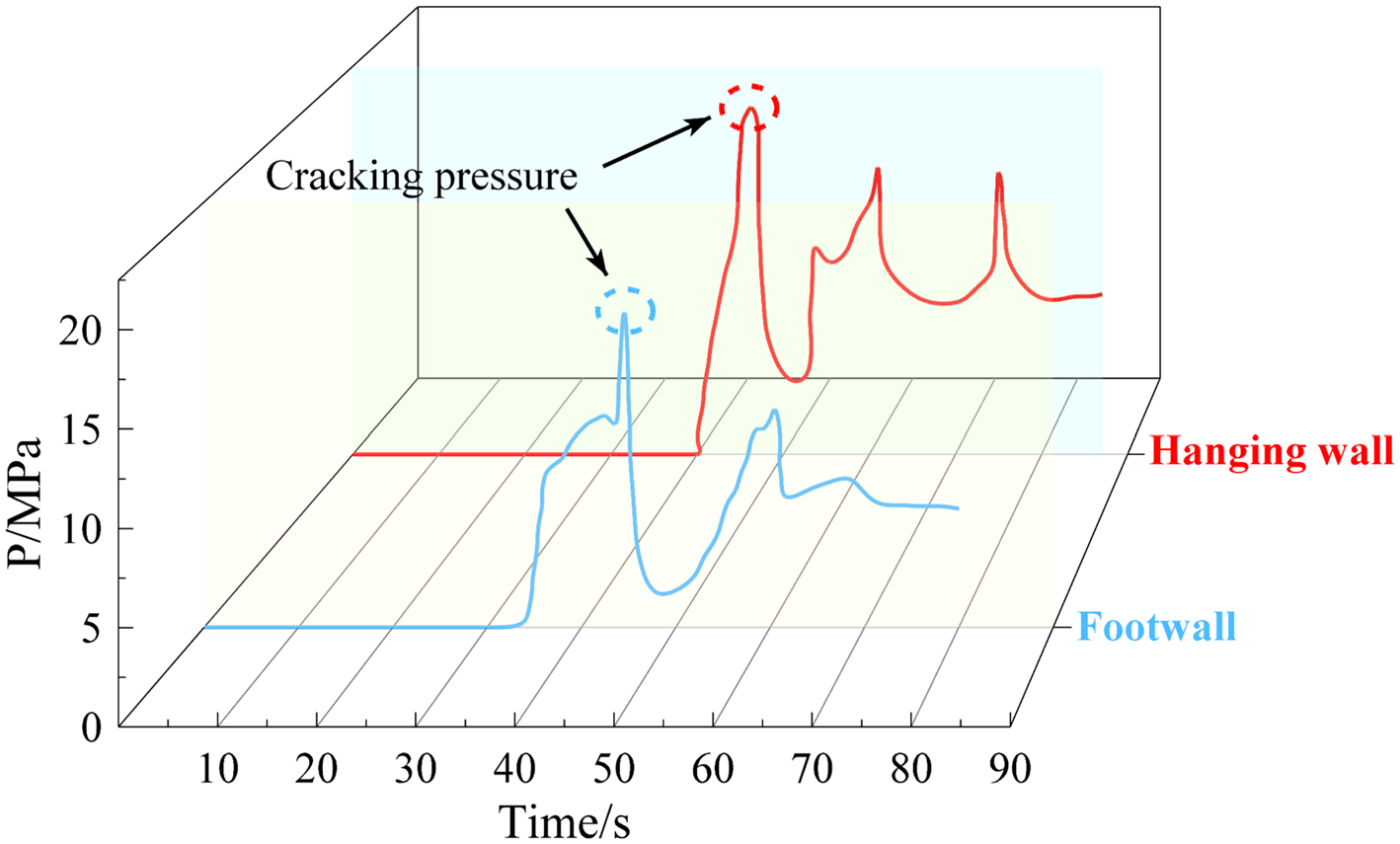
– 600 m elevation normal fault hanging wall and footwall crack initiation pressure.

Figure 13 shows the variation in water pressure over time during the hydraulic fracturing process. Since the variation in water pressure over time during the fracturing process is essentially the same for coal samples at − 400 m, − 600 m, and − 800 m, only the fracturing curve for coal at − 600 m is analyzed here for brevity. After activating the servo pressurization system, water was injected into the fracturing pipe. As the pressurized water gradually filled the borehole, the internal pressure began to rise. At 40 s, the water pressure surged rapidly, reaching a peak of 20.20 MPa at 46 s, when the first main fracture initiated in the hanging wall coal specimen. The pressure dropped abruptly due to fracture propagation but rebounded as newly formed fractures were filled with injected water, driving further crack extension until fractures penetrated the specimen surface. Due to the stress-induced compression of fractures, which creates significant fracture resistance, the residual water pressure remains around 9 MPa even after the coal sample is completely fractured. For the footwall coal specimen, activation of the servo pressurization system triggered water injection into the fracturing pipe. At 30 s, the water pressure began a rapid ascent, reaching its peak of 17.08 MPa by 44 s, when the first main fracture initiated in the footwall coal. Fracture propagation followed patterns similar to those observed in the hanging wall, with cyclic pressure fluctuations reflecting crack extension and filling. After full fracture penetration, residual water pressure stabilized near 6 MPa. The crack initiation pressure during hydraulic fracturing is higher in the hanging wall coal of the normal fault compared to the footwall coal. This may be due to the presence of the normal fault, which induces differences in the stress environment, leading to variations in the crack pressure of hydraulic fracturing. Moreover, even for coal at the same elevation, the breakdown pressure can still exhibit significant differences.

**Fig. 14:**
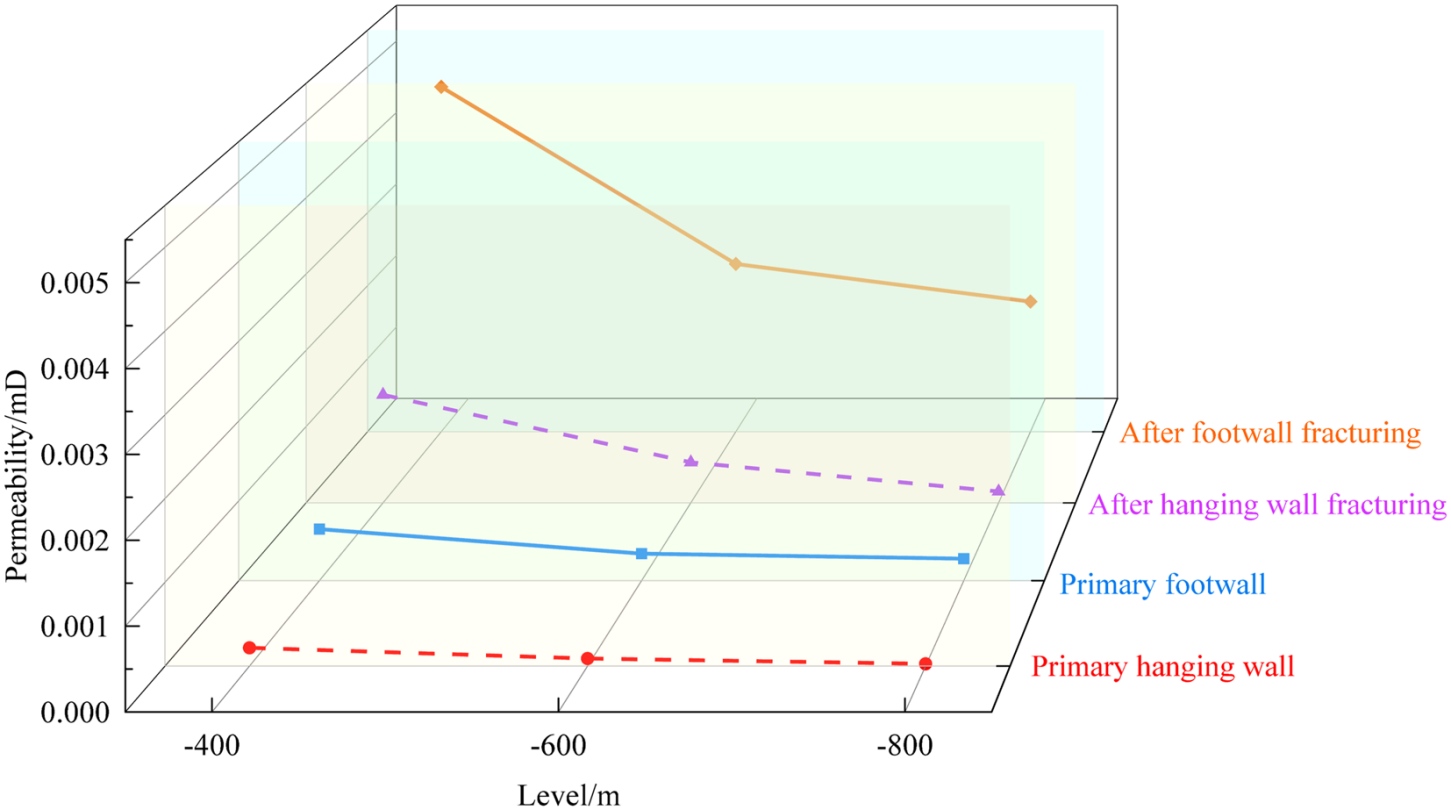
Comparison of permeability changes in coal before and after hydraulic fracturing in a normal fault.

Figure 14 shows the changes in gas permeability of coal in the hanging wall and footwall of a normal fault before and after hydraulic fracturing. The gas permeability after hydraulic fracturing refers to the permeability measured when the first primary fracture appears in the coal sample.

Under true triaxial stress conditions at − 400 m, − 600 m, and − 800 m, after hydraulic fracturing, the permeability of coal in the hanging wall of the normal fault increased to 0.001417 mD, 0.000531 mD, and 0.00014904 mD, respectively. Compared to the permeability before hydraulic fracturing, the permeability of coal in the hanging wall of the normal fault increased by 5.5 times, 4.9 times, and 4.52 times, respectively. For the coal in the footwall of the normal fault, the permeability increased to 0.00472 mD, 0.0022984 mD, and 0.001782 mD, respectively. Compared to the permeability before hydraulic fracturing, the permeability of coal in the footwall of the normal fault increased by 6.31 times, 5.8 times, and 5.48 times, respectively (Fig. 14). Guo et al.^20^ found that under high-pressure water injection, the permeability of coal can increase by 1.4 to 5.6 times, which is consistent with the experimental results presented in this study. During the high-pressure water injection process, the coal matrix surrounding the pores and fractures is subjected to compressive forces, leading to the expansion of the pores and fractures. When the fluid pressure in the fractures exceeds the rupture pressure of the coal matrix, new fractures may form in the fracture direction or connect with existing fractures, thereby enhancing the opening and connectivity of the internal fractures in the coal. This provides more pathways for gas flow and increases the permeability of the coal. Additionally, water injection also has a softening effect on the coal matrix^21^. As the pressure of the flowing gas increases, the degree of fracture opening in the softened coal further increases, resulting in a significant increase in gas flux within the fractures after fracturing, compared to before water injection^22^. This demonstrates a marked trend of increased permeability in the coal. After hydraulic fracturing, the permeability of coal in both the hanging wall and footwall of the normal fault significantly increased. Overall, the increase in permeability in the footwall coal is greater than that in the hanging wall coal. We believe this may be due to the presence of the Guodishan normal fault, which caused significant changes in the stress environment and mechanical properties of the hanging wall coal, thereby affecting the fracture propagation during hydraulic fracturing and the resulting increase in permeability.

**Fig. 15:**
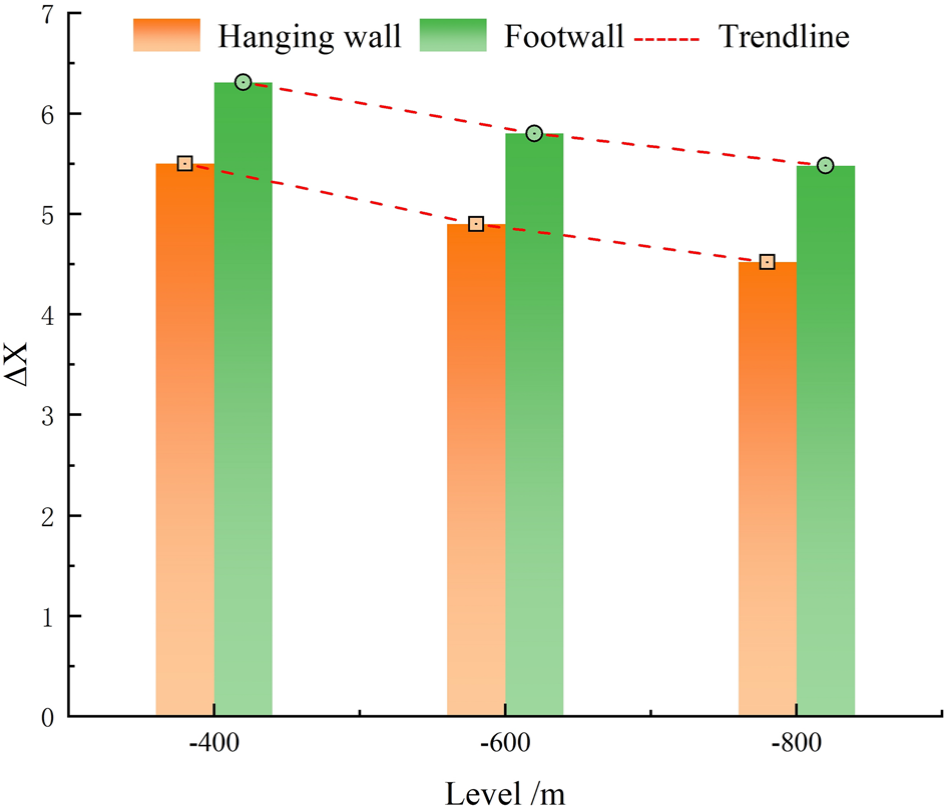
Variation in permeability increment after hydraulic fracturing.

Figure 15 shows the variation in permeability increment before and after hydraulic fracturing in the coal of the hanging wall and footwall, $\Delta X = \left( {X_{2} - X_{1} } \right)/X_{1}$, where *X*_*1*_ represents the initial permeability and *X*_*2*_ represents the permeability after fracturing. Regardless of whether it is the hanging wall or footwall, as the burial depth increases, the permeability increment gradually decreases. In the hanging wall, it decreases from 5.5 to 4.52, and in the footwall, it decreases from 6.31 to 5.48. After hydraulic fracturing, although the permeability of both the hanging wall and footwall coal significantly increased, the permeability of the footwall remains significantly higher than that of the hanging wall. Specifically, at − 400 m and − 600 m elevations, the permeability of the footwall is 3.33 times and 4.33 times higher than that of the hanging wall, respectively (Fig. 14). This indicates that as the burial depth increases, the permeability enhancement effect of hydraulic fracturing gradually weakens. This may be due to the higher in-situ stress in deeper coal seams, which results in a denser fracture structure, making it difficult for hydraulic fracturing to fully propagate fractures, thus reducing the effectiveness of permeability improvement. This difference suggests that, during gas extraction and gas outburst prevention, permeability enhancement measures in the footwall of normal faults may be more advantageous than in the hanging wall. Gas is more easily able to flow and be extracted in the footwall, while in the hanging wall, due to the lower permeability, more intensive permeability enhancement measures may be required to improve extraction efficiency and prevent coal and gas outburst accidents.

## Enhanced gas control technology for the Guodishan normal fault

### Gas control enhancement strategies for the hanging wall of the Guodishan fault

Affected by the Guodishan normal fault structure, the Pingmei Jiu Coal Mine and Pingmei Wu Coal Mine have implemented regional gas outburst prevention measures for the Ji_16-17_ coal seams in accordance with national regulations and local policies. Specifically, the Ji_15_ coal seam is mined first to protect the safe mining of the Ji_16-17_ coal seams. Based on the experimental results in Sections “Test device and scheme” and “Results and analysis”, the coal body in the hanging wall of the Guodishan normal fault exhibits low permeability, and its permeability enhancement effect is relatively poor. This may result in poor pressure relief effectiveness of protective layer mining methods in areas of the Guodishan normal fault’s hanging wall with drastic coal seam thickness variations and gas accumulation zones. Additionally, due to the unique evolutionary background of the Guodishan normal fault, the coal in the hanging wall has poor gas storage stability, with structural coal development. After the protective layer mining, while gas extraction drilling is carried out, additional permeability enhancement measures, such as hydraulic punching and hydraulic fracturing, need to be applied to achieve supplementary and reinforced gas extraction in areas with abnormal coal thickness and gas accumulation.

Considering the well-developed fractures in the coal masses within the Guodishan normal fault zone, as well as construction challenges and cost constraints, this study proposes a gas control strategy for the Ji_16-17_–22051 mechanized roadway in Jiu Coal Mine, located in the hanging wall. The approach integrates cross-seam borehole gas pre-drainage and anti-reflection technology of hydraulic punching to achieve efficient gas extraction and outburst mitigation in critical areas.

**Fig. 16:**
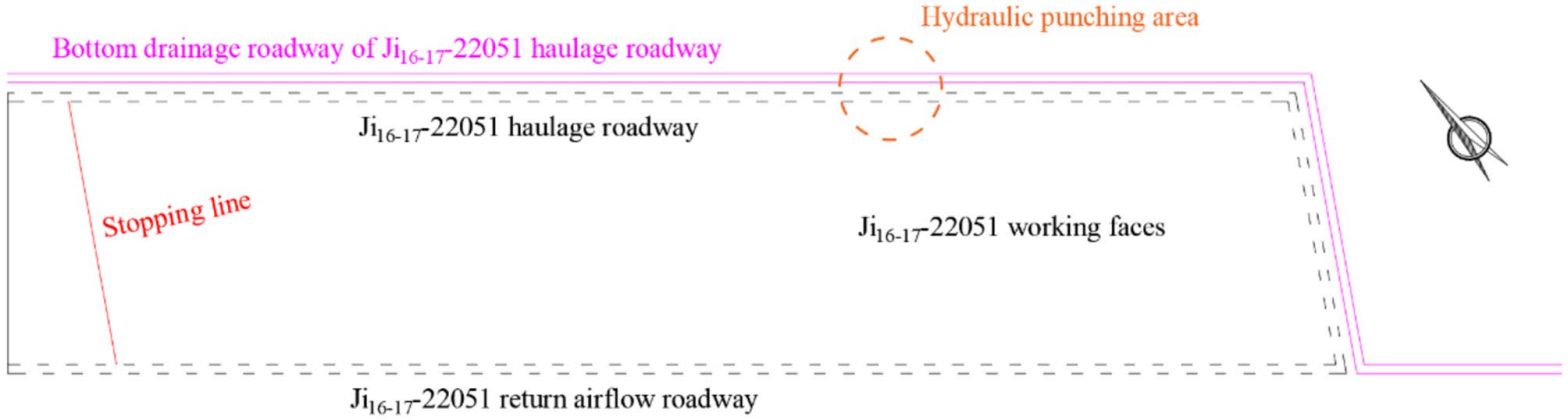
Schematic diagram of the Ji _16–17_-22051 working face.

The Ji_16-17_–22051 working face is located on the eastern part of the mining area extending downward from Ji-II. It borders the 22320 coal mining face of Wu Coal Mine to the south and an unmined solid coal pillar to the north. The working face has a designed strike length of 640 m, dip length of 140 m, mining height of 3.2 m, and a coal seam dip angle of approximately 15°, as shown in Fig. 16.

A bottom drainage roadway of Ji _16–17_-22051 haulage roadway arranged on the outer side of the Ji _16–17_-22051 haulage roadway. The bottom drainage roadway is offset 10 m outward from the haulage roadway and is located in the rock strata approximately 15 m below it. Upward-oriented cross-seam gas pre-drainage boreholes were drilled from the bottom drainage roadway, and permeability enhancement measure of hydraulic punching were implemented in thickened coal seam zones to ensure safe haulage roadway excavation.

#### Layout parameters of gas drainage and hydraulic punching boreholes

**Fig. 17:**
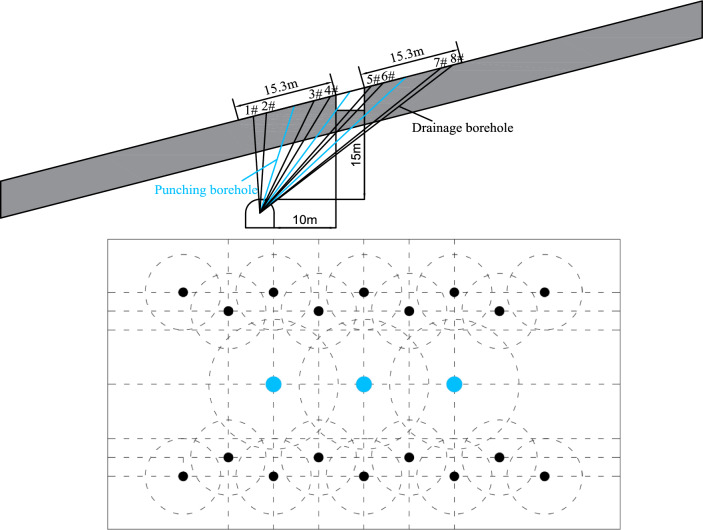
Schematic diagram of hydraulic punching borehole arrangement.

The Ji _16–17_-22051 haulage roadway is driven along the roof of the Ji _16–17_ coal seam, featuring a trapezoidal cross-section with a width of 4.6 m and height of 3.2 m. Gas pre-drainage boreholes were designed with a drainage radius of 2.5 m, while hydraulic punching boreholes adopted an influence radius of 5 m. In compliance with the "Regulations on the Prevention and Control of Coal and Gas Outbursts"^23^ the borehole layout covers a 15 m zone on both sides of the roadway. The borehole design is illustrated in Fig. 17.

#### Hydraulic punching process

Construction sequence: Drilling → Identifying coal seam location → Punching the entire coal section → The returned water becomes clear。

Drilling: prior to hydraulic punching borehole, drilling should be carried out according to the predetermined borehole diameter and angle. Detailed records of the waste slag discharge should be maintained, and the precise location of the coal seam should be determined.

Punching Borehole: after passing through the coal seam, replace the drill bit with a nozzle and advance it to the predetermined position within the coal seam. Set the emulsified liquid pump pressure, start the pump, and perform hydraulic punching. Then, gradually punch from the coal–rock interface into the deeper part of the borehole, while rotating and reciprocating the drill rod to facilitate slag discharge. After the initial punching, additional punching may be performed from the inner side outward using an advancing–retracting method until clear water returns. To prevent drill jamming and borehole blockage, control the punching speed or perform punching intermittently. However, during periods of significant coal discharge, drilling must not be halted; additional drill rods should be added when the coal discharge is low and the return water flow is normal. In the event of blockage, the water supply can be temporarily stopped, but drilling should continue to facilitate powder discharge. Ensure unobstructed drainage prior to punching to maintain a favorable working environment on site.

Punching Pressure: the initial punching pressure is set below 5 MPa. The pressure is then increased by 2 MPa every 5 min, with a maximum limit of 15 MPa.

Punching Duration: the hydraulic punching process lasts for 20 to 30 min from start to finish, or until more than 3 tons of coal have been discharged.

#### Evaluation of the hydraulic punching effect

To improve gas extraction performance at the working face, supplementary measures were adopted, including the use of hydraulic punching technology to enhance coal seam permeability and extending the extraction duration. These measures are intended to increase the permeability of the coal seam, thereby improving extraction efficiency and reducing both the gas pressure and gas content in the seam. After the completion of these hydraulic punching supplementary measures, the effectiveness in the evaluation area was assessed.

**Fig. 18:**
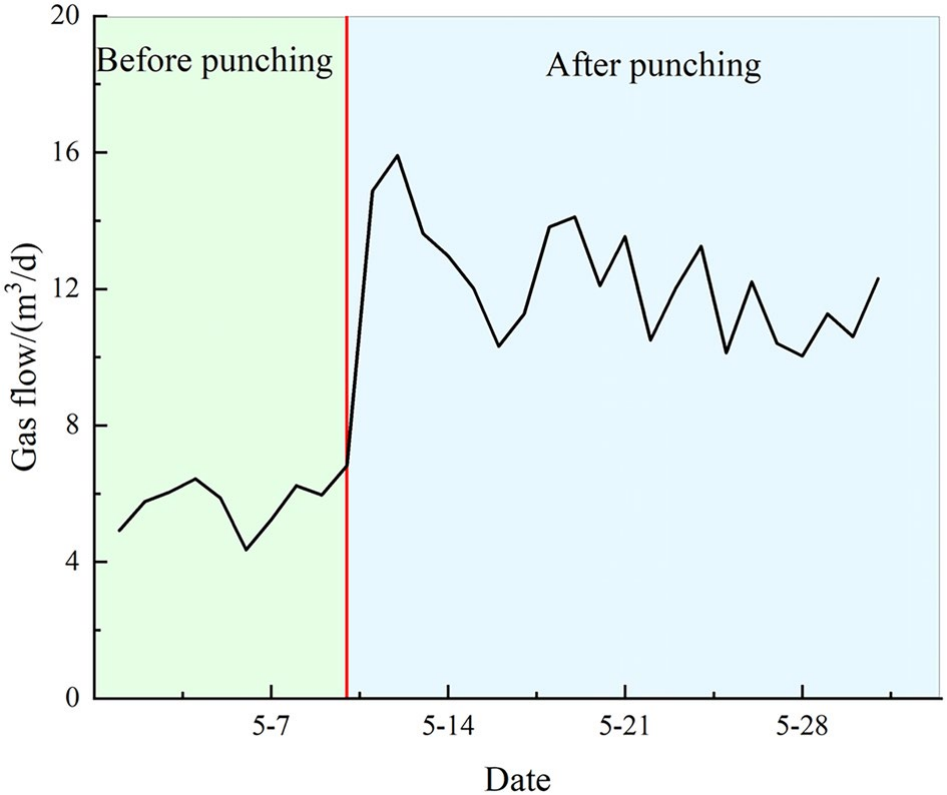
Variation in gas flow rate of extraction boreholes.

Boreholes #1 through #8 were connected to the gas meter to continuously monitor the gas extraction flow rate from a set of cross-seam gas pre-drainage boreholes before and after hydraulic punching, as shown in Fig. 18. Before hydraulic punching, the average net gas flow rate was 5.75 m^3^/d and the maximum net gas flow rate was 6.81 m^3^/d; after hydraulic punching, these values increased to an average of 12.25 m^3^/d and a maximum of 15.9 m^3^/d, representing increases of 2.13 times and 2.33 times, respectively. Clearly, after hydraulic punching, both the net gas flow rate and gas concentration improved significantly. Even 20 days after punching, a relatively high net gas extraction flow rate was maintained, demonstrating a remarkable depressurization and permeability enhancement effect.

In the hydraulic punching area of the Ji_16-17_ coal seam, the maximum participating gas content in the working face was 4.10 m^3^/t, and the maximum residual gas pressure was 0.355 MPa. Compared with the original gas content and pressure, these values were significantly reduced, meeting the safety standards for construction.

### Gas control enhancement strategies for the footwall of the Guodishan fault

Based on the experimental results in Sections “Test device and scheme” and “Results and analysis”, we believe that the coal in the footwall of the Guodishan normal fault exhibits better permeability, with more developed fractures. Based on the onsite geological conditions, Wu Coal Mine, located in the footwall of the Guodishan fault, is currently mining the Ji_16-17_ and Ji_15_ coal seams in the Jisanxiayan mining area and the Jisi mining area. The Jisi mining area is located near the Guodishan fault, and due to the presence of numerous secondary open fault structures of the Guodishan fault within the mining area, the regional gas content is relatively low. For example, in the Ji_15_-24090 working face, according to the regional outburst hazard prediction compiled by the mine, the Ji_15_ coal seam within a 20 m range outside the working face cut to the stop line is predicted to be a non-outburst hazard zone. Therefore, direct mining is feasible. However, during production, gas drainage through interlayer boreholes should be prioritized in the footwall to extract gas from the Ji_15_ coal seam, to ensure the safe mining of the main Ji_16-17_ coal seam.

## Conclusion

1. With an increase in confining pressure, the permeability of coal in both the hanging wall and footwall of the normal fault significantly decreases, exhibiting a nonlinear trend. The permeability shows a negative exponential relationship with confining pressure, with a curve fitting coefficient as high as 0.98. Moreover, as confining pressure increases, the permeability difference between the two sides of the fault gradually diminishes; under the same confining pressure, the permeability of the hanging wall is consistently lower than that of the footwall.
2. Under true triaxial stress conditions, the permeability of the coal in the hanging wall of the normal fault is generally lower than that in the footwall. This difference gradually increases with burial depth, with the permeability ratio between the footwall and hanging wall rising from 2.96 to 10.2 times. Additionally, as the burial depth increases, the permeability of the hanging wall coal decreases by a greater degree than that of the footwall coal.
3. Under true triaxial stress conditions, the crack initiation pressure for the coal in the hanging wall of the normal fault is higher than that in the footwall. After hydraulic fracturing, the permeability of the coal in both the hanging wall and footwall increases significantly, with the footwall showing a greater enhancement (5.48–6.31 times) compared to the hanging wall (4.52–5.5 times). However, as the burial depth increases, the permeability enhancement factors for both the hanging wall and footwall coal exhibit a gradually decreasing trend.
4. Based on the permeability characteristics of the coal in the hanging wall and footwall of the Guodishan normal fault, and taking into account technical challenges and construction costs, we propose the Guodishan Normal Fault Gas Enhancement and Control Technology. This technology involves implementing cross-layer gas pre-drainage combined with hydraulic punching permeability enhancement in areas of the hanging wall where the protective layer depressurization effect is inadequate, and applying cross-layer gas pre-drainage in areas of the footwall with unsatisfactory protective layer depressurization.

## Data Availability

The data used to support the findings of this study are available from the corresponding author upon request.
